# High-resolution quantification of stress perfusion defects by cardiac magnetic resonance

**DOI:** 10.1093/ehjimp/qyae001

**Published:** 2024-01-09

**Authors:** Cian M Scannell, Richard Crawley, Ebraham Alskaf, Marcel Breeuwer, Sven Plein, Amedeo Chiribiri

**Affiliations:** Department of Biomedical Engineering, Eindhoven University of Technology, Groene Loper 5, 5612 AE Eindhoven, The Netherlands; School of Biomedical Engineering and Imaging Sciences, King’s College London, London SE1 7EH, UK; School of Biomedical Engineering and Imaging Sciences, King’s College London, London SE1 7EH, UK; School of Biomedical Engineering and Imaging Sciences, King’s College London, London SE1 7EH, UK; Department of Biomedical Engineering, Eindhoven University of Technology, Groene Loper 5, 5612 AE Eindhoven, The Netherlands; School of Biomedical Engineering and Imaging Sciences, King’s College London, London SE1 7EH, UK; Leeds Institute of Cardiovascular and Metabolic Medicine, University of Leeds, Leeds LS2 9JT, UK; School of Biomedical Engineering and Imaging Sciences, King’s College London, London SE1 7EH, UK

**Keywords:** quantitative stress perfusion CMR, high resolution

## Abstract

**Aims:**

Quantitative stress perfusion cardiac magnetic resonance (CMR) is becoming more widely available, but it is still unclear how to integrate this information into clinical decision-making. Typically, pixel-wise perfusion maps are generated, but diagnostic and prognostic studies have summarized perfusion as just one value per patient or in 16 myocardial segments. In this study, the reporting of quantitative perfusion maps is extended from the standard 16 segments to a high-resolution bullseye. Cut-off thresholds are established for the high-resolution bullseye, and the identified perfusion defects are compared with visual assessment.

**Methods and results:**

Thirty-four patients with known or suspected coronary artery disease were retrospectively analysed. Visual perfusion defects were contoured on the CMR images and pixel-wise quantitative perfusion maps were generated. Cut-off values were established on the high-resolution bullseye consisting of 1800 points and compared with the per-segment, per-coronary, and per-patient resolution thresholds. Quantitative stress perfusion was significantly lower in visually abnormal pixels, 1.11 (0.75–1.57) vs. 2.35 (1.82–2.9) mL/min/g (Mann–Whitney *U* test *P* < 0.001), with an optimal cut-off of 1.72 mL/min/g. This was lower than the segment-wise optimal threshold of 1.92 mL/min/g. The Bland–Altman analysis showed that visual assessment underestimated large perfusion defects compared with the quantification with good agreement for smaller defect burdens. A Dice overlap of 0.68 (0.57–0.78) was found.

**Conclusion:**

This study introduces a high-resolution bullseye consisting of 1800 points, rather than 16, per patient for reporting quantitative stress perfusion, which may improve sensitivity. Using this representation, the threshold required to identify areas of reduced perfusion is lower than for segmental analysis.

## Graphical Abstract

**Graphical Abstract:**
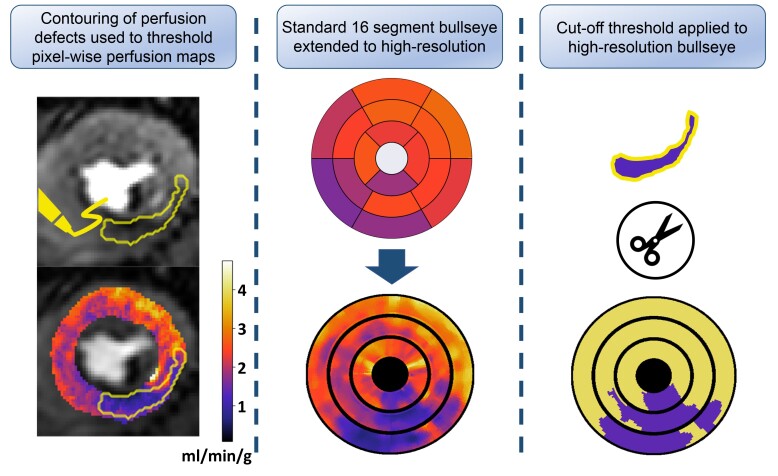

## Introduction

Stress perfusion cardiac magnetic resonance (CMR) can detect obstructive coronary artery disease (CAD) with high diagnostic accuracy,^1^ is suitable to guide the revascularization of patients with stable angina,^2^ and is useful in the evaluation of myocardial ischaemia in a wide range of conditions.^3–5^ Although the clinical use of stress perfusion CMR is expanding, it is still relatively underutilized, as accurate visual interpretation of the images depends on the availability of highly trained operators. The quantification of perfusion has the potential to make the assessment user-independent and thus more widely available.^6^

In recent years, there have been technical advancements in both the acquisition and the analysis of the data that are primed to aid clinical translation and improve the utility of quantitative stress perfusion CMR. These include work to improve the spatial resolution^7^ and to increase the spatial coverage of the ventricle,^8–10^ facilitated by motion-compensated compressed sensing reconstructions.^11^ These developments allow the identification of transmural gradients in perfusion and ensure that all coronary territories are covered, as well as simplifying the acquisition and analysis. In particular, the high spatial resolution is a key advantage of CMR over positron emission tomography (PET) and single-photon emission computed tomography.^12^ Dual-sequence acquisitions have also been developed to correct for the saturation of the arterial input function (AIF) and enable the quantification of myocardial blood flow (MBF) in a streamlined process.^13,14^ More recently, fully automated solutions for quantifying perfusion CMR are being integrated into the clinical workflow, with Artificial Intelligence (AI)–based image analysis^15–17^ and AI-based quantification.^18,19^ However, none of these works have addressed the reporting and interpretation of the quantitative values.

Despite the availability of pixel-wise perfusion maps, to date, diagnostic and prognostic studies report global or 16 segmental [defined by the American Heart Association (AHA)^20^] MBF values,^21–23^ eliminating the benefits of the high spatial resolution. The 16-segment model also adds further challenges to the reporting of perfusion defects in a standardized way for the typical case where a perfusion defect affects multiple segments to varying degrees. Additionally, the perfusion images should be interpreted in the context of the late gadolinium enhancement (LGE) images in order to distinguish ischaemic regions from scar. This is also limited by the current approach of assigning only one classification to each segment, which does not allow the identification of areas of peri-infarct ischaemia in a part of a segment.

Consequently, existing stress MBF thresholds validated to detect myocardial ischaemia (range between 1.8 and 2.0 mL/min/g) do not reflect the MBF within a perfusion defect, but rather the averaged MBF across a myocardial segment, which may include normally perfused and ischaemic pixels. The MBF specifically within perfusion defects is unknown. As the mean MBF in a segment will average the normal and abnormal pixels within that segment, the segment-wise cut-off values previously validated are likely to be higher than those to be used at a higher spatial resolution. Due to their elevated values, if segment-wise thresholds are applied directly to a pixel-wise analysis, then the higher threshold will likely result in more false-positive pixels and thus an overestimation of the ischaemic burden. Additionally, there is a potential loss of diagnostic information, as dichotomizing 16 AHA segments allows the reporting of ischaemic burden only in multiples of 6.25%. More importantly, the use of discrete segments reduces the sensitivity to detect mild ischaemia or smaller perfusion defects that occupy less than half of a segment.

Therefore, the aim of this study was two-fold. First, to derive a pixel-wise MBF threshold to detect stress perfusion defects and to compare this with segmentally derived thresholds, and secondly, based on the pixel-wise assessment, to extend the traditional segment-wise interpretation to a high-resolution representation for reporting quantitative stress perfusion CMR.

## Methods

### Population

Thirty-four patients who underwent stress perfusion CMR scans, 28 of which have been previously reported^24^ as having visually apparent stress perfusion defects and 6 patients with visually normal perfusion, were retrospectively identified. All patients gave written informed consent (regional ethics committees: 15/NS/0030 and 18/ES/0115), and patients were instructed to refrain from caffeine for 24 h prior to the CMR examination.

### CMR acquisition

All CMR examinations were performed using a clinical 3 T system (Achieva TX, Philips Healthcare, Best, The Netherlands) equipped with a 32-channel cardiac phased array coil. Perfusion image acquisition used a previously described dual-sequence implementation with electrocardiogram triggering.^13^ High-resolution images were acquired in three short-axis slices covering the left ventricle (LV; basal, mid, and apical) in addition to the low-resolution AIF slice during adenosine-induced hyperaemia (140–210 μg/kg/min, depending on the response to stress). The contrast agent was a 0.075 mmol/kg dose of gadobutrol (Gadovist, Bayer, Berlin, Germany), injected intravenously at 4 mL/s, followed by a 25 mL saline flush at the same injection rate.

### Visual assessment

Perfusion defects were first identified by visual assessment of the dynamic stress perfusion images. The myocardium was manually segmented, and perfusion defects were manually contoured on the dynamic with maximal observed myocardial contrast. The analysis was performed jointly by two experienced operators.^24^ The rest of the images were not analysed and the presence or absence of LGE was not considered.

### MBF quantification

The dynamic perfusion images were retrospectively corrected for respiratory motion, and a pixel-wise MBF was quantified using a fully automated pipeline,^4,5^ employing a previously described Fermi function–constrained deconvolution implementation.^6^ The concentration of contrast was approximated from the signal intensity values with a relative signal enhancement conversion^25^ and was used for the kinetic parameter quantification with the AIF from the low-resolution short saturation time slices and the myocardial tissue response curves from the standard slices. The automated processing included segmentation of the myocardium, identification of the LV blood pool for the AIF, and detection of the right ventricular (RV) insertion points. The RV insertion points were used to combine pixel-wise perfusion values in the 16 AHA segments.

### High-resolution bullseye representation

**Figure 1:**
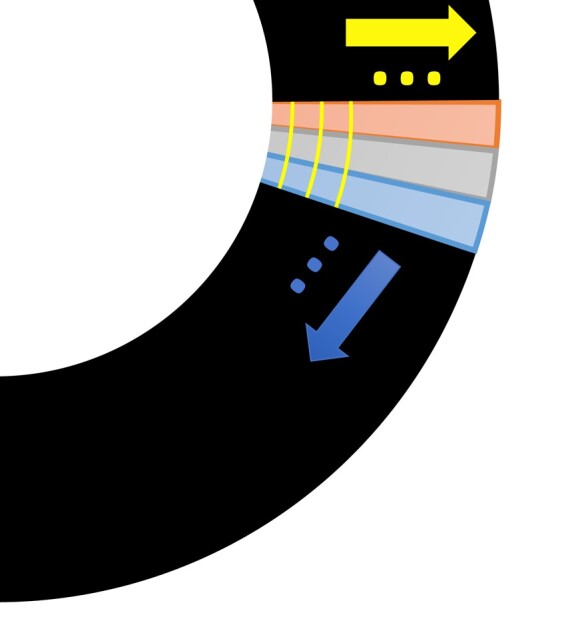
An illustration of a segment of the myocardium showing the transmural layers from the subendocardium to the subepicardium (left to right) and different angular regions (top to bottom).

An extension to the 16-segment AHA model was developed, similar to a previously used model.^26,27^ Each of the three slices is divided into 10 transmural layers from the subendocardium to the subepicardium and 60 angular positions (illustrated in *Figure 1*). This results in 600 grid points per slice and a total of 1800 per patient. Algthough it depends on the slice location and cardiac phase and can differ significantly from patient to patient, this leads to around one pixel per high-resolution segment point, at a typical imaging resolution of 2 × 2 mm^2^. Perfusion maps are interpolated to this grid using cubic b-spline interpolation. This high-resolution representation should retain more of the diagnostic information and allow a comparison between different CMR acquisitions (e.g. cine and LGE images) by standardizing the irregular shape of the myocardium, without sacrificing resolution. The regular shape of the sampled grid also facilitates a more simplified filtering of the data. In this work, a median filter smoothing with a filter size of 5 is applied, to reduce the effect of outliers on the analysis, while preserving sharp transitions at the borders of perfusion defects.

### Data analysis

Perfusion defect segmentations from the visual analysis were superimposed on the pixel-wise perfusion maps after motion correction, and the perfusion defect burdens were calculated as the sum perfusion defect area divided by the sum myocardial area and expressed as a percentage. The correlation of manually segmented perfusion defects and quantified defects was assessed using the Pearson’s correlation coefficient, and an agreement was examined using the Bland–Altman analysis. Overlap between the automatically quantified perfusion defects and the manually segmented perfusion defects was measured by using the Dice score coefficient (DSC).^28^

MBF was not normally distributed and was summarized as median (interquartile range) and compared between groups using a Mann–Whitney *U* test. An optimal MBF threshold to detect pixels that lie within the visual perfusion defects, as manually segmented, was derived using the Youden index from a receiver operator characteristic (ROC) curve analysis. MBF thresholds were computed on the pixel by AHA segment, coronary territory, and patient level. An AHA segment was considered positive for reduced perfusion if more than half of the segment lay within a visually identified perfusion defect. A coronary territory was considered positive if it had a least one positive segment, and a patient was considered positive if any coronary territory was positive. The MBF value for an AHA segment was taken as the average MBF of all pixels in that segment, and the MBF value for a coronary territory was the average of the two lowest segments in that territory, as previously validated.^29^ The MBF value for a patient was the average MBF of all pixels of that patient. The statistical significance was set at *P* = 0.05.

## Results

**Figure 2:**
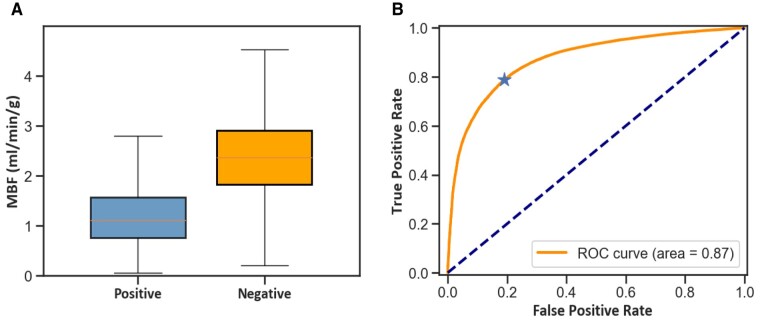
(*A*) Box plots showing the distribution of pixel-wise stress MBF within areas positive and negative for visually apparent perfusion defects, respectively. (*B*) The ROC analysis shows the diagnostic performance of automated pixel-wise CMR perfusion for the detection of pixels with visually reduced perfusion, with an optimal threshold for detecting 1.72 mL/min/g (marked with *).

The median stress MBF of all pixels was 2.1 (1.42–2.74) mL/min/g. MBF was significantly lower within segmented perfusion defects [1.11 (0.75–1.57) mL/min/g] when compared with outside perfusion defects [2.35 (1.82–2.9) mL/min/g; *P* < 0.001]. The distributions of MBF values within and outside of areas positive for perfusion defects are shown in *Figure 2A*. The optimal MBF value to detect pixels within segmented perfusion defects was 1.72 mL/min/g, which yielded an area under the curve (AUC) of 0.87 with the ROC curve shown in *Figure 2B*. This corresponds to a pixel-wise accuracy of detecting perfusion defects of 79.4% with a sensitivity rate of 78.9% and a specificity rate of 80.9%.

For the segment-wise analysis, the optimal cut-off value from the ROC curve analysis was 1.92 mL/min/g, giving an AUC of the ROC curve of 0.87, an accuracy rate of 78.1% (425/544 myocardial segments), a sensitivity rate of 75.3%, and a specificity rate of 86.1%. On the level of the coronary territories, the optimal cut-off value was 1.91 mL/min/g, yielding an AUC of 0.91, an accuracy rate of 81.4% (83/102 coronary territories), a sensitivity rate of 89.5.1%, and a specificity rate of 73.3%. On the whole patient level, the optimal cut-off value was 2.61 mL/min/g, yielding an AUC of 0.98, an accuracy rate of 91.2% (31/34 patients), a sensitivity rate of 100%, and a specificity rate of 92.9%.

**Figure 3:**
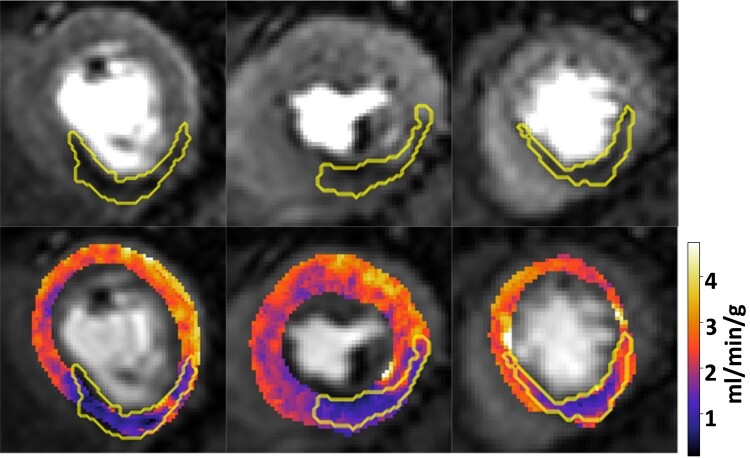
(Top) Stress perfusion images with visually apparent perfusion defects in the territory of the right coronary artery and the superimposed motion-corrected perfusion defect segmentation. (Bottom) Pixel-wise perfusion maps (in units of mL/min/g) with superimposed perfusion defect segmentations. Pixel-wise maps additionally demonstrate evidence of subendocardial ischaemia in the basal and mid segments of the left anterior descending coronary artery territory, which were not appreciated on visual assessment.

**Figure 4:**
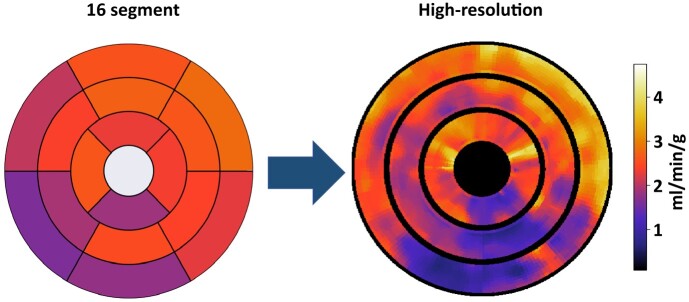
An extension of the standard AHA 16-segment representation to the high-resolution 1800-point (600 per slice) bullseye plot proposed in this paper. The same patient shown in *Figure 3* is shown.

**Figure 5:**
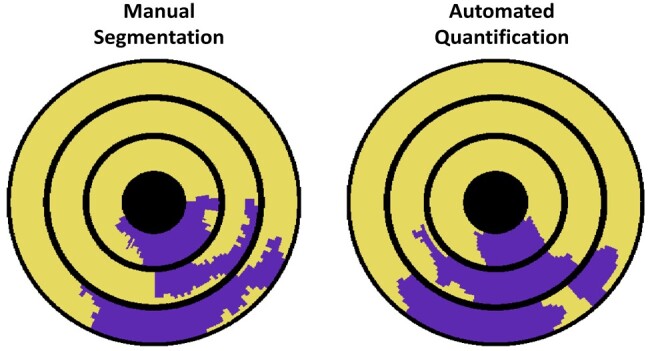
Manual segmentation converted to the high-resolution bullseye representation (left) in comparison with the automatically quantified thresholded high-resolution bullseye representation (right) for the same patient shown in *Figures 3* and *4* is shown.

An example case is presented in *Figure 3*, showing the manually segmented perfusion defect (top row) and the quantitative perfusion maps with the segmentations overlaid (bottom row). For this same case, *Figure 4* shows the extension of the standard AHA 16-segment representation to a high-resolution 1800-point bullseye plot, and *Figure 5* shows a comparison between the manually segmented and the automatically quantified high-resolution perfusion defect map. A pixel-wise MBF threshold of 1.72 mL/min/g was applied to the high-resolution bullseye plots to identify areas of reduced perfusion, creating the perfusion defect maps in *Figure 5*.

**Figure 6:**
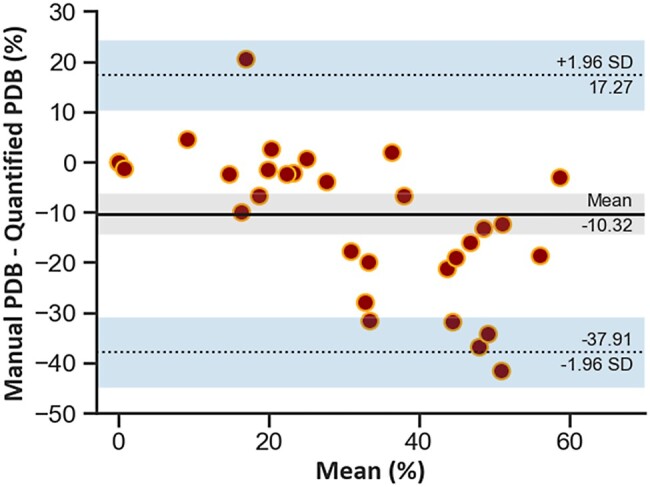
Bland–Altman plot demonstrates a proportional bias, with an increasing effect of quantitative analysis on the perfusion defect burden as the burden increases. The dotted lines represent the limits of agreement, and the shaded regions are 95% confidence intervals.

**Figure 7:**
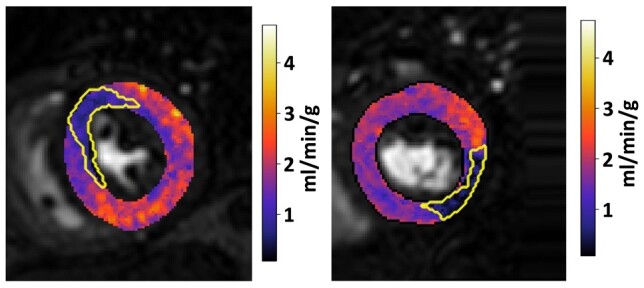
Two example slices from different patients with widespread reduced perfusion where the visual segmentation (overlaid) underestimates the perfusion defect.

The median (25th percentile, 75th percentile) DSC overlap between manual defect segmentations and regions of reduced automated quantitative MBF (<1.72 mL/min/g) was 0.68 (0.57, 0.78). A strong positive correlation between the manually segmented and the quantitative perfusion defects was observed (*r* = 0.83). While the Bland–Altman analysis showed a good agreement, with an overall mean bias of −10.3%, there was a proportional bias (*β* = 0.52, *P* < 0.001; *Figure 6*). For smaller perfusion defect burdens, there was a very good agreement between the manually and the automatically quantified defects, but for larger defects, the manual segmentation underestimates the burden when compared with the automated quantification. *Figure 7* shows two example slices that illustrate this point. Here, the quantitative maps show widespread reductions in perfusion, but the visual assessment (overlaid) identifies only the most prominent area of the defects. This is in line with previous work showing that with more extensive perfusion defects, in the case of multi-vessel CAD, the ischaemic burden may be underestimated by visual assessment.^30^

## Discussion

This work introduced an approach to standardizing the reporting of quantitative stress perfusion CMR on the pixel-wise level, facilitated by fully automated AI-based image processing. Despite the fact that stress perfusion CMR allows for the quantification of pixel-wise MBF, data are typically averaged over the whole myocardium or myocardial segments. Therefore, information is lost on the locality and spatial distribution of perfusion defects. A related study recently demonstrated the use of smaller segments and found that this improved diagnostic accuracy,^31^ but this is the first study to directly evaluate perfusion maps at the pixel-wise level. Similarly, our study found that stress MBF can detect pixels within visually apparent myocardial perfusion defects with high accuracy. The optimal MBF threshold to detect stress perfusion defects was lower than segmentally derived thresholds, leading to the conclusion that previously validated thresholds would not be appropriate to detect pixel-wise perfusion defects and would overestimate ischaemic burden. The pixel-wise MBF threshold allowed the pixel-wise assessment of perfusion defects, and it was found that this agreed well with visual assessment for lower burdens. However, a further validation of such an approach is warranted with a consideration of the demonstrated discordance of visual and automatically quantified perfusion defects. Furthermore, we have shown (e.g. in *Figure 4*) that the pixel-wise MBF assessment leads to larger differences between positive and negative regions by avoiding the smoothing effect caused by the averaging of positive and negative pixels within segments (*Figure 4*).

On a practical level, our results demonstrate the potential to move away from segment-wise interpretations and towards a pixel-wise quantification and interpretation of perfusion to take full advantage of the high resolution offered by CMR, potentially increasing the sensitivity in discriminating ischaemic subendocardial regions from adjacent well-perfused subepicardial regions.^32^ High-resolution perfusion assessment can lead to a more accurate quantification of ischaemic burden. Ischaemic burden is an important prognostic marker^33^ that is used to guide revascularization or medical therapy in patients with CAD.^34^ Furthermore, for patients with epicardial CAD, treatment decisions are often made by equating AHA segments to coronary vessels, but there are large anatomical variations in coronary anatomy, and therefore, an assumption of coronary territory using the 16-segment AHA model can be inaccurate if not correlated directly with the individual coronary anatomy. High-resolution quantitative maps could also allow a more individualistic understanding of ischaemia through the study of the pixel-wise distribution of perfusion defects without assigning them into generic presumed coronary territories.^35^ As such, better prognostic decisions could be made by providing interventional cardiologists with more information from a high-resolution representation of the degree and distribution of ischaemia.

As well as improving the clinical assessment of ischaemia from epicardial CAD, the high-resolution mapping offers the potential for a new generation of applications of stress perfusion CMR. The myocardium can be further subdivided into subendocardial and subepicardial layers to aid the identification of coronary microvascular dysfunction (CMD).^3^ On top of this, the pixel-wise distribution of ischaemia may prove useful to distinguish CMD from CAD.

In the context of guiding treatment, the relationship between both myocardial ischaemia from epicardial CAD and myocardial viability from previous infarction is complex. Visual assessment of myocardial infarction scarring is often used to help clinicians grade myocardial viability and assess whether apparent perfusion defects are genuinely ischaemic in nature. Emerging techniques that allow a quantitative segmentation of an LGE scar could facilitate a move away from making ‘yes’ or ‘no’ decisions on whether a segment is viable or not. In theory, this could then be combined with our quantitative perfusion maps to distinguish true ischaemia from fixed perfusion defects caused by an infarction scar. The combination of these analyses could be facilitated by our standardized high-resolution bullseye and could improve accuracy in the identification of peri-infarct ischaemia and quantification of ischaemic burden in patients with complex CAD. In the future, these diagnostic/prognostic decisions could be aided by further AI models. This high-resolution assessment of the location, extent, and spatial distribution of disease will be critical information for such models, which are unlikely to succeed if restricted to AHA values. The standardized bullseye representation makes the data more suitable as input to machine learning models than varying sizes and shapes of the myocardium, as the anatomical differences are normalized, allowing the models to focus directly on the tissue characteristics. This may ease the development of AI-based diagnostic and prognostic models.

This work presents a promising proof-of-concept approach to report pixel-wise MBF and quantify perfusion defects. However, further validation is required to prove its clinical utility and overcome some of its potential drawbacks. For example, as more information is generated, work is required to efficiently integrate these additional data into a clinical report without increasing the complexity for the reporting physician. Reporting typically focuses on classifying the 16 AHA myocardial segments, but our proposed approach allows for a more detailed assessment. Recently, an atlas for a standardized reporting of PET myocardial perfusion studies has been introduced by the American Society for Nuclear Cardiology,^36^ and a similar initiative should be undertaken for stress perfusion CMR quantitative MBF values. High-resolution bullseye plots should be included in the report, similar to polar map plots in nuclear cardiology. The high spatial resolution should allow separation between focal and diffuse diseases, and a similar bullseye representation could be made using an AI-enabled segmentation of LGE images^37^ to allow a combined assessment of perfusion and scar and the identification of peri-infarct ischaemia. Therefore, classifications can be made per vessel with a distinction among suspected epicardial ischaemia, microvascular ischaemia, and scar. Still, many patients who will not benefit from the intervention are referred for revascularization, and the more detailed stress CMR reporting, as outlined above, may address this by reducing referrals of patients unlikely to benefit from an invasive procedure because their ischaemia is microvascular in nature, or they lack sufficient viable tissue to benefit.

This study considered only stress MBF data, with thresholds derived from a small cohort of retrospectively identified patients, with a high prevalence of disease, investigated using a single scanner; as such, the findings may not be transferable to other patient populations or different acquisition settings. In particular, the generalizability of stress perfusion CMR values across systems is not guaranteed. This may limit the applicability of these results to other data, and in general, there is a need for the community and vendors to standardize the analysis to encourage clinical adoption.

The MBF thresholds used visual assessment as a comparison. This is a limitation, as visual interpretation necessitates significant expertise, training, and confidence on the part of the reporter to make accurate assessments. Furthermore, visual interpretation is subject to inherent intra-observer and inter-observer variability. A more robust analysis would compare our approach against a quantitative reference standard, such as fractional flow reserve. Based on this promising proof-of-concept study, a larger-scale prospective evaluation vs. invasive physiological indices in a multi-centre setting should be considered as future work.

## Conclusion

This work proposes a high-resolution assessment of quantitative perfusion CMR at the pixel-wise level, introducing a standardized approach to reporting the high-resolution data based on a bullseye plot of 1800 points per patient. When interpreting perfusion maps at a pixel-wise level, stress MBF can detect pixels within visually apparent myocardial perfusion defects with high accuracy, paving the way for a more detailed quantification of the extent and severity of myocardial ischaemia. The pixel-wise MBF threshold in detecting perfusion defects is lower than segmentally derived thresholds previously validated to detect myocardial ischaemia, and thus, generalizing segmentally derived stress MBF thresholds to a pixel-wise analysis may result in a substantial overestimation of the ischaemic burden.

**Conflict of interest:** None declared.

## Data Availability

The data underlying this article cannot be shared publicly due to ethical restrictions. The data can be shared on reasonable request to the corresponding author.

## Lead author biography

Dr Cian M. Scannell studied Mathematical Sciences at University College Cork (Ireland), where he graduated with first-class honours in 2016. He then moved to King’s College London to undertake his master’s and PhD degrees as part of the Centre for Doctoral Training in Medical Imaging. Here, he worked on a thesis entitled ‘Automated quantitative analysis of first-pass myocardial perfusion magnetic resonance imaging data’. In 2021, he received his PhD and then continued for a further year as a postdoctoral researcher funded by the Wellcome/EPSRC Centre for Medical Engineering at King’s College London with a project on the development of robust and generalizable models for clinical integration. In 2022, he was appointed as an assistant professor with the Medical Image Analysis group in the Department of Biomedical Engineering at the Eindhoven University of Technology. Mandatory photo credit: Vincent van den Hoogen.
